# The gift of novelty: repeat-robust *k*-mer-based estimators of mutation rates

**DOI:** 10.1093/bioinformatics/btag234

**Published:** 2026-07-07

**Authors:** Haonan Wu, Paul Medvedev

**Affiliations:** Department of Computer Science and Engineering, The Pennsylvania State University, University Park, PA 16802, United States; Department of Computer Science and Engineering, The Pennsylvania State University, University Park, PA 16802, United States; Department of Biochemistry and Molecular Biology, The Pennsylvania State University, University Park, PA 16802, United States; Huck Institutes of the Life Sciences, Pennsylvania State University, University Park, PA 16802, United States

## Abstract

**Motivation:**

Estimating mutation rates between evolutionarily related sequences is a central problem in molecular evolution. Due to the rapid expansion of datasets, modern methods avoid costly alignment and instead compare sketches of sets of constituent *k*-mers. While these methods perform well on many sequences, they are not robust to highly repetitive sequences such as centromeres.

**Results:**

We present three new estimators that are robust to the presence of repeats. The estimators are applicable in different settings, depending on whether count information is available from zero, one, or both sequences. We evaluate our estimators empirically using highly repetitive alpha satellite sequences. Each estimator performs best within its class, and our strongest estimator outperforms all other tested estimators.

**Availability and implementation:**

Our software is open-source and freely available at https://github.com/medvedevgroup/Accurate_repeat-aware_kmer_based_estimator.

## 1 Introduction

Estimating mutation rates between evolutionarily related sequences has long been a central problem in molecular evolution, originating well before the advent of large-scale genomics. Early quantitative methods concentrated on amino acid substitution rates, such as the PAM matrices introduced by Dayhoff (1969) and the BLOSUM matrices developed by Henikoff and Henikoff (1992). These methodologies, along with later profile-based hidden Markov models (Durbin 2013) continue to serve as the benchmark when high-quality alignments can be obtained.

Nevertheless, the rapid growth of sequencing has made computationally intensive alignment-based pipelines increasingly infeasible in the modern era. As a result, alignment-free methods that characterize sequences using low-cost summary statistics have become essential (Song *et al.* 2014, Zielezinski *et al.* 2017, Rathore and Kashyap 2026). Most of these techniques are based on sketches of *k*-mer spectra. Widely used tools such as Mash (Ondov *et al.* 2016) and Skmer (Sarmashghi *et al.* 2019), along with more recent sketch-adjusted approaches including Sylph (Shaw and Yu 2025) and FracMinHash-based methods (Hera *et al.* 2023, 2024, 2025, Shaw and Yu 2023, Hera and Koslicki 2025, Wu *et al.* 2025), enable rapid construction of whole-genome phylogenies, efficient metagenomic screening, and the estimation of millions of pairwise point-mutation rates in minutes rather than days.

Nearly all alignment-free approaches are derived based on the assumption that most *k*-mers above a certain *k*-mer size (e.g. k≥19) occur only once in a sequence. However, recent advances in sequencing technology are leading to more abundantly available highly repetitive sequences. For example, the recent telomere-to-telomere human assembly contains fully assembled chromosome centromeres, which are alpha satellite DNA composed of 171-bp monomers that are further arranged into higher-order repeats (Logsdon *et al.* 2024). Unfortunately, most estimators are not robust to repeat-rich sequences and methods to analyze the mutation rates between such sequences remain in their infancy.

We can categorize the space of *k*-mer-based estimators based on the type of information they use. In the absence of repeats, it suffices to consider the set of *k*-mers present in the sequences and ignore their occurrence counts. We call these type of estimators as Presence–Presence, because they rely on presence/absence information for both the source string *s* and the mutated string *t*. Such estimators are especially useful in the setting where occurrence counts are not readily available, such as raw sequencing data. In the presence of repeats, however, occurrence counts become an important signal. In the Presence–Count setting, an estimator is restricted to presence/absence information for *s* but is allowed to use occurrence counts of *t*. This can occur if for example *s* is unassembled sequencing data while *t* is an assembly. The Count–Count setting is the most powerful, allowing the estimator to use information about counts in both *s* and *t*, but is limited to applications such as when both *s* and *t* are assembled.

The widely known Mash estimator falls in the Presence–Presence category, but, as we show in this paper, is not repeat-robust. There are two repeat-robust *k*-mer-based estimators that we are aware of. The first is from our previous work (Wu *et al.* 2025), which, as we will describe in Sec. 7, falls roughly in between the Presence–Presence and the Presence–Count setting. The second is a weighted-intersection-based estimator, which falls in the Count–Count setting. This is a natural estimator that has been mentioned in Rhie *et al.* (2020).

In this paper, we present three new estimators (Table 1): ${\hat{q}}_{\text{pp}}$, for the Presence–Presence setting, ${\hat{q}}_{\text{pc}}$, for the Presence–Count setting, and ${\hat{q}}_{\text{cc}}$, for the Count–Count setting. One of our main insights is that the number of newly created *k*-mers is more sensitive to the presence of repeats than the number of *k*-mers that remain shared (or, as reflected in the title, we treat *novel k*-mers as a *gift* to make use of).

**Table 1:** Our contributed estimators.

Name	Knowledge of *k*-mers in *s*	Knowledge of *k*-mers in *t*	Formula
${\hat{q}}_{\text{pp}}$	Presence/absence	Presence/absence	$\frac{\left|sp(t)\setminus sp(s)\right|}{L}$
${\hat{q}}_{\text{pc}}$	Presence/absence	Counts	$\sum_{\tau \in sp(t)\setminus sp(s)}\frac{\mathrm{occ}(\tau ,t)}{L}$
${\hat{q}}_{\text{cc}}$	Counts	Counts	${\hat{q}}_{\text{pc}}+{\left(1-{\hat{r}}_{\text{pc}}\right)}^{k-1}\cdot \frac{{\hat{r}}_{\text{pc}}}{3L}\cdot \sum_{\tau \in sp(s)}occ(\tau ,s)\cdot d_{1}(\tau ,s)$

Here, *s* in arbitrary string with L=|s|−k+1; *t* is the result of applying a mutation process to *s* with substitution rate *r*; $d_{1}(\tau ,s)$ is the number of *k*-mers in the spectrum of *s* that are at a Hamming distance of one to τ. We use *q* as shorthand for $1-{\left(1-r\right)}^{k}$, e.g. an estimator $\hat{q}$ implicitly defines $\hat{r}=1-{\left(1-\hat{q}\right)}^{1/k}$.

We evaluate our estimators empirically using various types of sequences, including the alpha satellite centromeric region from a human chromosome. We evaluate estimator error across a wide range of mutation rates and *k*-mer values. Moreover, we demonstrate how our estimators can be combined with FracMinHash sketching without systematically effecting bias. Ultimately, each of our estimators performs best in their class, with ${\hat{q}}_{\text{cc}}$ outperforming estimators in all classes. Finally, we show how our estimators can be used on real data by applying them to compute the ANI (Average Nucleotide Identity), which is widely used to measure genomic distance in taxonomic analysis.

## 2 Preliminaries

Let *s* be a string and let k>0 be the *k*-mer size. We let |s| denote the number of nucleotides in *s*. We assume in this paper that |s|≥k. We use *L* to denote the number of *k*-mers in *s*, i.e. L=|s|−k+1. Let sp(s) be the set of all distinct *k*-mers in *s*, also called the *spectrum* of *s*. Let occ(τ,s) denote the number of copies of *k*-mer τ in string *s*. Let $d_{i}(\tau ,s)$ denote the number of *k*-mers in sp(s) with Hamming distance *i* to *k*-mer τ. The Jaccard similarity between two sets *A* and *B* is $J(A,B)≜\frac{\left|A\cap B\right|}{\left|A\cup B\right|}$.

We will consider the simple substitution mutation model as in previous work (Blanca *et al.* 2022). Given a parameter 0≤r≤1 and a string *s*, the model generates an equal-length string where, independently, the character at each position is unchanged from *s* with probability 1−r and changed to one of the three other nucleotides with probability r/3.

Our goal in this paper is to estimate the mutation rate *r* given observations about *s* and *t*. We assume that, at a minimum, we can observe *L*, sp(s), and sp(t). Depending on the category of the estimator, we may also observe occ(τ,s) and/or occ(τ,t), for all τ.

As a shorthand notation, we define $q≜1-{\left(1-r\right)}^{k}$; intuitively, *q* is the probability that a *k*-mer is mutated. In this work and others, estimators for *r* are usually derived by first obtaining an estimator $\hat{q}$ for *q* and then computing the estimator $\hat{r}$ using the inverse formula $\hat{r}=1-{\left(1-\hat{q}\right)}^{1/k}.$ We take the same approach in this paper, where we will explicitly define an estimator for *q* and leave the definition of *r* implicit using the above formula.

The *bias* of an estimator $\hat{q}$ is defined as $E[\hat{q}]-q$. An unbiased estimator will on average return the correct value. In the case of mutation rate estimators, it is usually easier to derive the bias of $\hat{q}$ rather than $\hat{r}$. Even when there is a closed-form expression for the bias of $\hat{q}$, it does not lead to a closed-form expression for the bias of $\hat{r}$, because $\hat{r}$ is not linear in $\hat{q}$. In this paper, we will derive the bias of our *q* estimators when possible. However, we will ultimately rely on experimental results to judge the bias of our estimators.

In this paper, we will take the *method-of-moments* approach to derive our estimators (Wasserman 2013). We first decide on a random variable to observe, e.g. $I^{\text{pp}}=|sp(s)\cap sp(t)|$. We then derive its expectation (possibly approximating it under some assumptions), e.g. $E[I^{\text{pp}}]\approx L(1-q)$. We then take the observed value (denoted by $I_{\text{obs}}^{\text{pp}}$), plug it into the expectation formula, and solve the formula for *q* to get the estimate. In our example, $\hat{q}$ is the solution to the equation $I_{\text{obs}}^{\text{pp}}=L(1-\hat{q})$, which is $\hat{q}=1-I_{\text{obs}}^{\text{pp}}/L$.

## 3 The Presence–Presence setting

In this section, we consider the setting where the only thing we know about *s* and *t* are their spectra and *L*, i.e. no count information. We propose the following estimator:


$${\hat{q}}_{\text{pp}}=\frac{N^{\text{pp}}}{L},$$

where $N^{\text{pp}}=|sp(t)\setminus sp(s)|$ is the number of new distinct *k*-mers generated when *s* mutates into *t*. We will use the notation whereby a superscript of “pp” indicates the Presence–Presence setting, “pc” indicates the Presence–Count setting, and “cc” indicates the Count–Count setting. In this section, we explain how ${\hat{q}}_{\text{pp}}$ is derived and compare it to other known estimators for the Presence–Presence setting. We do not attempt to derive the bias of ${\hat{q}}_{\text{pp}}$, as it is technically complicated.

Consider the k-span model, which is to assume that

1. *s* does not contain any *k*-mer that occur more than once, and
2. new *k*-mers generated after mutations are distinct from each other and from the *k*-mers in *s*.

Let us define $I^{\text{pp}}≜|sp(s)\cap sp(t)|$ and use the shorthand J≜J(sp(s),sp(t)). In the *k*-span model, we have that $E[I^{\text{pp}}]=L(1-q)$ and the method-of-moments approach gives the estimator:


(1) $$\hat{q}=\frac{L-I_{\text{obs}}^{\text{pp}}}{L}.$$

This estimator was introduced in Wu *et al.* (2025). Furthermore, in this model, $J=I^{\text{pp}}/(2L-I^{\text{pp}})$ and as a result, we have that $\frac{1-J}{1+J}=\frac{L-I^{\text{pp}}}{L}$. Thus, we can equivalently write:


(2) $$\hat{q}=\frac{1-J_{\text{obs}}}{1+J_{\text{obs}}}.$$

This is an improved version of the Mash estimator (Ondov *et al.* 2016), described in Sarmashghi *et al.* (2019) and in Appendix A.6 of Belbasi *et al.* (2022). Finally, in this model, we have that $N^{\text{pp}}+I^{\text{pp}}=L$, so, we can equivalently write:


(3) $$\hat{q}=\frac{N_{\text{obs}}^{\text{pp}}}{L},$$

which corresponds the definition of our new estimator ${\hat{q}}_{\text{pp}}$. The three versions of $\hat{q}$ have the same derivation and are algebraically equivalent in the *k*-span model. However, when applied to data violating the *k*-span assumptions, we will see that three versions produce different results and ${\hat{q}}_{\text{pp}}$ outperforms the others (Sec. 7).

The intuition for ${\hat{q}}_{\text{pp}}$ is based on considering what happens when assumption 1 is violated, i.e. there are repeats. Let τ be a *k*-mer with at least two occurrences in *s* and let ν be a *k*-mer that appears exactly once in *s*. Every mutation leads to a new *k*-mer in $N^{\text{pp}}$, regardless of whether it happens in an occurrence of τ or ν. On the other hand, a mutation in one of the copies of τ does not effect $I^{\text{pp}}$ while a mutation in ν increases $N^{\text{pp}}$ by one. This makes Equations (1) and (2), which rely on $I^{\text{pp}}$, less accurate than Equation (3).

## 4 The Presence–Count setting

In this section, we consider the Presence–Count setting and derive our estimator ${\hat{q}}_{\text{pc}}$ and its bias. Given two strings *s* and *t*, we define


$$N^{\text{pc}}≜\sum_{\tau \in sp(t)\setminus sp(s)}occ(\tau ,t).$$

Intuitively, $N^{\text{pc}}$ is the number of new *k*-mers generated when *s* mutates into *t*. We will derive ${\hat{q}}_{\text{pc}}$ by applying a method-of-moments approach to $N^{\text{pc}}$. Let us first define


$$R^{j}(\tau ,s)≜d_{j}(\tau ,s){\left(1-r\right)}^{k-j}{\left(\frac{r}{3}\right)}^{j}\;\text{and}\;R(\tau ,s)≜\sum_{j=1}^{k}R^{j}(\tau ,s)$$

$R^{j}(\tau ,s)$ is the probability that τ mutates to a *k*-mer that is in *s* and has a Hamming distance of *j* to τ. R(τ,s) is the probability that τ mutates to a *k*-mer that is in *s* but is different from τ. Note that, we defined $R^{j}$ and *R* in terms of *r* since it is visually clearer, but we can equivalently think of them as functions of *q*. We can now derive $E[N^{\text{pc}}]$.

Lemma 1. Let s be a string and let t be a string generated from s using the mutation process parameterized by r. Then,

(4) $$E[N^{\text{pc}}]=Lq-\sum_{\tau \in sp(s)}occ(\tau ,s)R(\tau ,s).$$

**Proof.** 

Let HD(τ,ν) denote the Hamming distance between *k*-mers τ and ν. For 1≤i≤L, let $s_{i}$ be the *k*-mer starting at position *i* of *s*. Let $X_{i}$ be an indicator random variable representing the event that $s_{i}$ mutated to a *k*-mer that does not appear in *s*, i.e. $t_{i}\notin s$. We can express $N^{\text{pc}}$ as a sum $X_{i}$s as follows.

$$N^{\text{pc}}=\sum_{\tau \in sp(t)\setminus sp(s)}occ(\tau ,t)=\sum_{i=1}^{L}1[t_{i}\notin sp(s)]=\sum_{i=1}^{L}X_{i},$$

where 1 is the indicator function for an event. Then,

$$\begin{array}{l} E[N^{\text{pc}}]=\sum_{i=1}^{L}\text{Pr}[X_{i}=1]\\ =\sum_{i=1}^{L}\left(1-\text{Pr}[X_{i}=0]\right)\\ =L-\sum_{i=1}^{L}\sum_{\tau \in sp(s)}\text{Pr}[t_{i}=\tau ]\\ =L-\sum_{i=1}^{L}\left(\text{Pr}[t_{i}=s_{i}]+\sum_{\tau \in sp(s)\setminus s_{i}}\text{Pr}[t_{i}=\tau ]\right)\\ =L-\sum_{i=1}^{L}\left(1-q+\sum_{\tau \in sp(s)\setminus s_{i}}\text{Pr}[t_{i}=\tau ]\right)\\ =Lq-\sum_{i=1}^{L}\sum_{\tau \in sp(s)\setminus s_{i}}\text{Pr}[t_{i}=\tau ]\\ =Lq-\sum_{i=1}^{L}\sum_{j=1}^{k}\sum_{\begin{array}{c} \tau \in \;\text{sp}(s)\setminus s_{i}\\ \;s.\;t.\\ HD(\tau ,t_{i})=j \end{array}}\mathrm{Pr}[t_{i}=\tau ]\\ =Lq-\sum_{i=1}^{L}\sum_{j=1}^{k}d_{j}(s_{i},s){\left(1-r\right)}^{k-j}{\left(r/3\right)}^{j}\\ =Lq-\sum_{i=1}^{L}R(s_{i},s)=Lq-\sum_{\tau \in sp(s)}occ(\tau ,s)R(\tau ,s) \end{array}$$

□

A straightforward method-of-moments estimator based on Equation (4) would be to observe the value of $N^{\text{pc}}$ (denoted as $N_{\text{obs}}^{\text{pc}}$), let $f(r)=Lq-\sum_{\tau \in sp(s)}occ(\tau ,s)R(\tau ,s)$, and use numerical methods to solve the equation $N_{\text{obs}}^{\text{pc}}=f(r)$ for *r*. However, there is no guarantee for the uniqueness of the solution. Furthermore, it would be time-consuming and superfluous to compute R(τ,s) for all τ.

Instead, we derive an estimator based on an approximation. Consider the two terms of Equation (4). The first term is the expected number of positions whose *k*-mer mutated. This may over-count $N^{\text{pc}}$, and the second term corrects this by accounting for the possibility that a position mutates but to something that is already in *s*. As we expect the first term to dominate, we approximate $E[N^{\text{pc}}]\approx Lq$, leading to the estimator:


$${\hat{q}}_{\text{pc}}=\frac{N_{\text{obs}}^{\text{pc}}}{L}.$$

Unlike ${\hat{q}}_{\text{pp}}$, this formula accounts for the possibility that two occurrences of a *k*-mer in *s* mutate to the same new *k*-mer in *t*. The bias of ${\hat{q}}_{\text{pc}}$ follows immediately from Lemma 1:

Theorem 1. The bias of ${\hat{q}}_{\text{pc}}$ is

$$E[{\hat{q}}_{\text{pc}}]-q=-\sum_{i=1}^{k}\sum_{\tau \in sp(s)}\frac{\mathrm{occ}(\tau ,s)R^{i}(\tau ,s)}{L}.$$

Note that the bias is always negative, meaning that, on average, ${\hat{q}}_{\text{pc}}$ underestimates the true value. Moreover, the bias is a sum of *k* negative terms, which leads to the intuition of reducing the bias by including one of the terms inside the estimator itself—something we pursue in the next section.

## 5 The Count–Count setting

In this section, we first present a novel Count–Count estimator ${\hat{r}}_{\text{cc}}$ and then describe an alternative Count–Count estimator proposed by Rhie *et al.* (2020) in the context of assembly quality assessment.

### 5.1 New estimator ${\hat{q}}_{\text{cc}}$

Let us build on ${\hat{q}}_{\text{pc}}$ by trying to include the first term of the bias (i.e. $-\sum_{\tau }occ(\tau ,s)R^{1}(\tau ,s)/L$) into the estimator itself. With the method-of-moments approach, we would need to solve the following equation for *r*:


(5) $$N_{\text{obs}}^{\text{pc}}=Lq-{\left(1-r\right)}^{k-1}\cdot \frac{r}{3}\cdot \sum_{\tau \in s}occ(\tau ,s)\cdot d_{1}(\tau ,s).$$

Unfortunately, we cannot solve this equation analytically and we cannot guarantee that a numerical method would produce a unique solution. Instead, we will first compute the ${\hat{r}}_{\text{pc}}$ estimator and then plug into the right hand side of Equation (5). Our estimator is then defined as:


$${\hat{q}}_{\text{cc}}=\frac{N_{\text{obs}}^{\text{pc}}}{L}+{\left(1-{\hat{r}}_{\text{pc}}\right)}^{k-1}\cdot \frac{{\hat{r}}_{\text{pc}}}{3L}\cdot \sum_{\tau \in sp(s)}occ(\tau ,s)\cdot d_{1}(\tau ,s)$$

While this approach of plugging in one estimator in order to derive another one makes it challenging to prove anything, we will see that it performs extraordinarily well in practice.

To compute ${\hat{q}}_{\text{cc}}$, we need to compute $d_{1}(\tau ,s)$ for all τ∈sp(s). We do this in a straightforward two-pass algorithm using a hash table, resulting in runtime linear in *L*. There are more advanced ways of doing this (e.g. using suffix arrays), but we were not concerned with optimizing runtime since it was below one second.

### 5.2 Estimator mentioned in Rhie *et al.* (2020)

Rhie *et al.* (2020) mention an estimator that we recapitulate here to show how it fits in our framework. It is designed in the spirit of Equation (1) by relying on the intersection size but also integrating count information. Consider the size of the weighted intersection between the *k*-mers of *s* and *t*:


$$I^{\text{cc}}≜\sum_{\tau \in sp(s)\cup sp(t)}\min(\mathrm{occ}(\tau ,s),\mathrm{occ}(\tau ,t)).$$

Let us assume that when a mutation occurs, the new *k*-mer is not in *s*. Before any mutations, $I^{\text{cc}}=L$. When a *k*-mer τ mutates to another *k*-mer $\tau^{\prime }$, it increases $\mathrm{occ}(\tau^{\prime },t)$ by one and decreases occ(τ,t) by one. Since $\tau^{\prime }$ is not in *s*, $\mathrm{occ}(\tau^{\prime },t)$ does not contribute anything to $I^{\text{cc}}$. Therefore, the only effect of a mutation on $I^{\text{cc}}$ is to decrease it by one. By linearity of expectation, we can add the probability of this happening for each of the *L* (non-distinct) *k*-mers and get that $E[I^{\text{cc}}]\approx L(1-q)$. The method-of-moments approach then gives the estimator:


$${\hat{q}}_{\text{wi}}≜1-I^{\text{cc}}/L.$$

## 6 Combination with sketching

A major reason why many estimators are based on *k*-mers rather than on the full sequences is that it makes them easily amenable to sketching. Sketching is a powerful technique that can make it possible to quickly compute all-pairs estimates on large datasets (Ondov *et al.* 2016). A *FracMinHash sketch* $sp_{\theta }(s)$ of a sequence *s* is defined as the subset of sp(s) that includes only the *k*-mers that map below a pre-defined threshold θ, under a fixed random hash function (Hera *et al.* 2023).

In this section, we will present a modification of ${\hat{q}}_{\text{pp}}$, ${\hat{q}}_{\text{pc}}$ and ${\hat{q}}_{\text{cc}}$ that work on data sketched with FracMinHash. Instead of observing $N^{\text{pp}}$ or $N^{\text{pc}}$, we now only observe them restricted to the sketched *k*-mers. Formally, let us define:


$$N_{\theta }^{\text{pp}}=|sp_{\theta }(t)\setminus sp_{\theta }(s)|\mathrm{and}N_{\theta }^{\text{pc}}=\sum_{\tau \in sp_{\theta }(t)\setminus sp_{\theta }(s)}occ(\tau ,t).$$

Intuitively, we expect each of these quantities to decrease by a factor of θ relative to their non-sketched versions. Based on this intuition, we define ${\hat{q}}_{\text{pp}}^{\theta }$ and ${\hat{q}}_{\text{pc}}^{\theta }$ as ${\hat{q}}_{\text{pp}}^{\theta }=\frac{N_{\theta }^{\text{pp}}}{\theta L}$ and ${\hat{q}}_{\text{pc}}^{\theta }=\frac{N_{\theta }^{\text{pc}}}{\theta L}$. Formalizing our intuition, we prove that sketching does not effect the bias.

Theorem 2. The biases of ${\hat{q}}_{\text{pp}}^{\theta }$ and ${\hat{q}}_{\text{pc}}^{\theta }$ are

$$E[{\hat{q}}_{\text{pp}}^{\theta }]-q=E[{\hat{q}}_{\text{pp}}]-q\;\text{and}\;E[{\hat{q}}_{\text{pc}}^{\theta }]-q=E[{\hat{q}}_{\text{pc}}]-q.$$

**Proof.** 

First, we show that $E[N_{\theta }^{\text{pc}}]=\theta \cdot E[N^{\text{pc}}]$. As before, let $X_{i}$ be an indicator random variable representing the event that $s_{i}$ mutated to a *k*-mer that does not appear in *s*. Let $Y_{\tau }$ be a binary random variable and it is 1 if *k*-mer τ hashes to less than θ. By the linearity, we have

$$\begin{array}{cc} E[N_{\theta }^{\text{pc}}] & =\sum_{i=1}^{L}\text{Pr}[Y_{s_{i}}=1\cap X_{i}=1]=\sum_{i=1}^{L}\text{Pr}[Y_{s_{i}}]\cdot \text{Pr}[X_{i}=1]\\ & =\sum_{i=1}^{L}\theta \cdot \text{Pr}[X_{i}=1]=\theta \cdot E[N^{\text{pc}}] \end{array}$$

Then,

$$E[{\hat{q}}_{\text{pc}}^{\theta }]-q=E\left[\frac{N_{\theta }^{\text{pc}}}{\theta \cdot L}\right]-q=\frac{E[N^{\text{pc}}]}{L}-q=E[{\hat{q}}_{\text{pc}}]-q$$

The proof for the bias of ${\hat{q}}_{\text{pp}}$ is similar and is omitted. □

Because ${\hat{q}}_{\text{pc}}^{\theta }$ shows the same bias as ${\hat{q}}_{\text{pc}}$, we use the same idea of Sec. 5.1 to obtain a stronger estimator ${\hat{q}}_{\text{cc}}^{\theta }$ by partially correcting the bias of ${\hat{q}}_{\text{pc}}^{\theta }$, i.e.


$${\hat{q}}_{\text{cc}}^{\theta }={\hat{q}}_{\text{pc}}^{\theta }+{\left(1-{\hat{r}}_{\text{pc}}^{\theta }\right)}^{k-1}\cdot \frac{{\hat{r}}_{\text{pc}}^{\theta }}{3L}\cdot \sum_{\tau \in sp(s)}occ(\tau ,s)\cdot d_{1}(\tau ,s).$$

Note that $\sum_{\tau \in s}occ(\tau ,s)\cdot d_{1}(\tau ,s)/L$ needs to be precomputed prior to sketching. It does not increase the space needed for the sketch as it becomes just a single constant that needs to be stored.

Because of the variance introduced by sketching, the observed quantity can exceed θL and then any of our three estimators can exceed 1. We therefore cap both estimators so that they never return >1.

## 7 Empirical results

We aim to evaluate the accuracy of our three novel estimators in relation to each other as well as to the other estimators mentioned in this paper. We also show an application to real data, where our estimators can be used to predict the ANI between various genomes.

### 7.1 Datasets

We use four different base sequences with various levels of repetitiveness, summarized in Table S1, available as supplementary data at *Bioinformatics* online. We use these sequences as representative examples spanning different levels of repetitiveness. Our main text evaluation is focused on the D-hardest sequence, which is a 100-kb-long sequences of alpha satellite DNA extracted from the human T2T chr21 centromere. We use a value of k=30 for our evaluation on this sequence, as consistent with previous analyses (Wu *et al.* 2025), leading to 3,987 distinct 30-mers. Over 70% of the distinct *k*-mers in D-hardest occur more than once and its *k*-mers have on average at least one other *k*-mer at a Hamming distance of one. D-hardest violates both assumption 1 (i.e. because it has repeats) and assumption 2 (i.e. because it has pairs of *k*-mers with small Hamming distances, a *k*-mer can mutate into one that is already in *s*). Since the differences between the estimators are more pronounced on this sequence, our main text focuses on D-hardest. The results on the three other sequences are presented in Section A (available as supplementary data at *Bioinformatics* online); they are all consistent with our findings on D-hardest but with less pronounced differences on less repetitive sequences.

### 7.2 Evaluation metrics

We focus our evaluation on the estimators’ accuracy, measuring both their bias and variance. We do not perform a runtime or memory analysis because they each complete in less than a second in total on all of the four datasets and use negligible memory.

**Figure 1:**
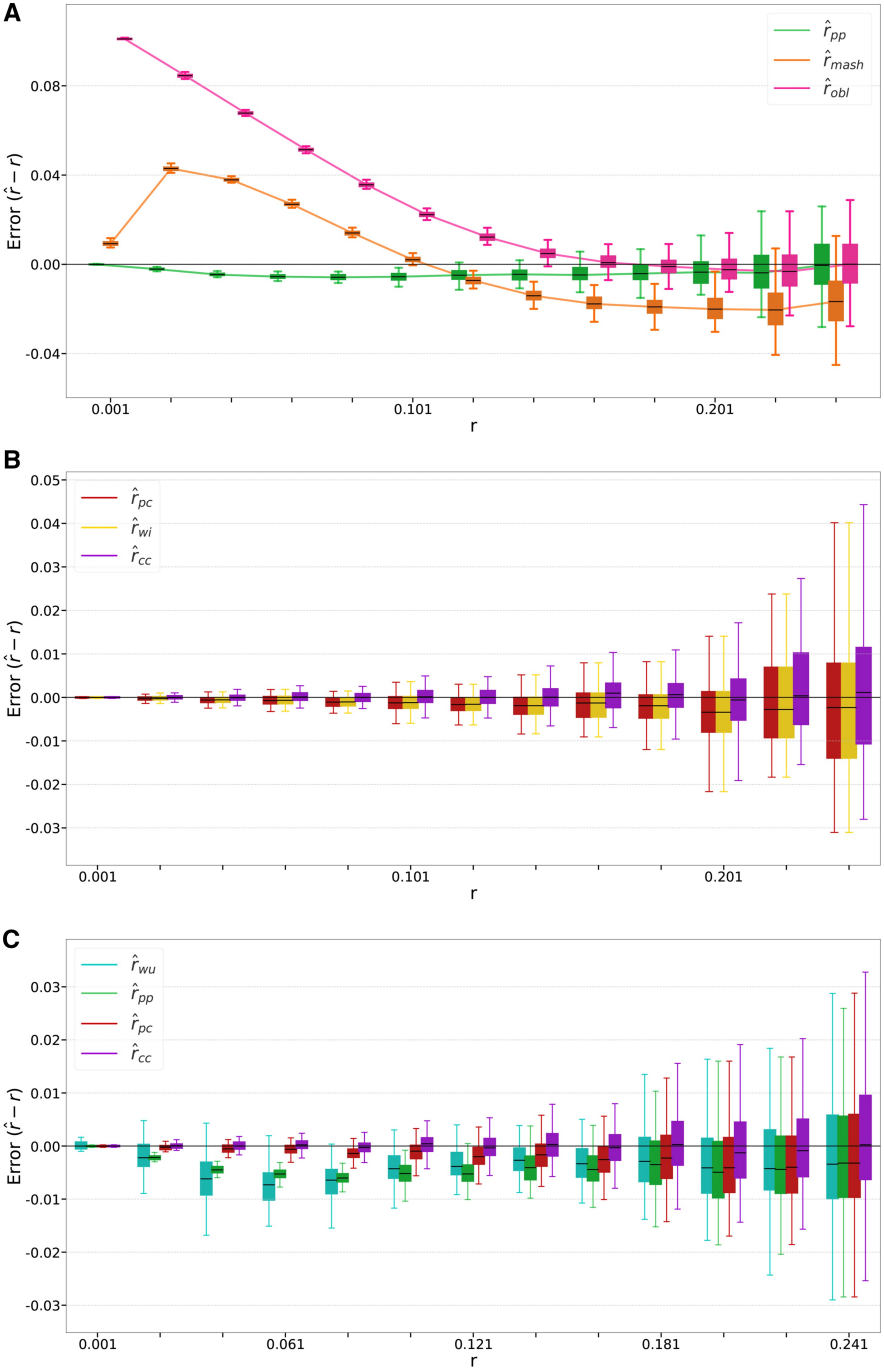
Comparison of estimator accuracy on D-hardest, with k=30 and mutation rate *r* from 0.001 to 0.251 with step size 0.02. For each value of *r*, we show box plots over 100 mutation replicates. (A) Comparison of ${\hat{r}}_{\text{pp}}$ against other presence–presence estimators. (B) Comparison of ${\hat{r}}_{\text{pc}}$ against ${\hat{r}}_{\text{wi}}$ and ${\hat{r}}_{\text{cc}}$. (C) Comparison of our three novel estimators against each other and against the ${\hat{r}}_{\text{wu}}$ estimator of Wu *et al*. (2025).

First, we benchmark each estimator on the four datasets using their default values of *k*, i.e. the values chosen in Wu *et al.* (2025) as most suitable for their analysis. We vary the mutation rate *r* from 0.001 to 0.251 and we show the distribution of $\hat{r}$ for 100 mutation-process simulation replicates for each *r* (e.g. Fig. 1). These experiments give a fine-grained separate view of the bias and variance.

**Figure 2:**
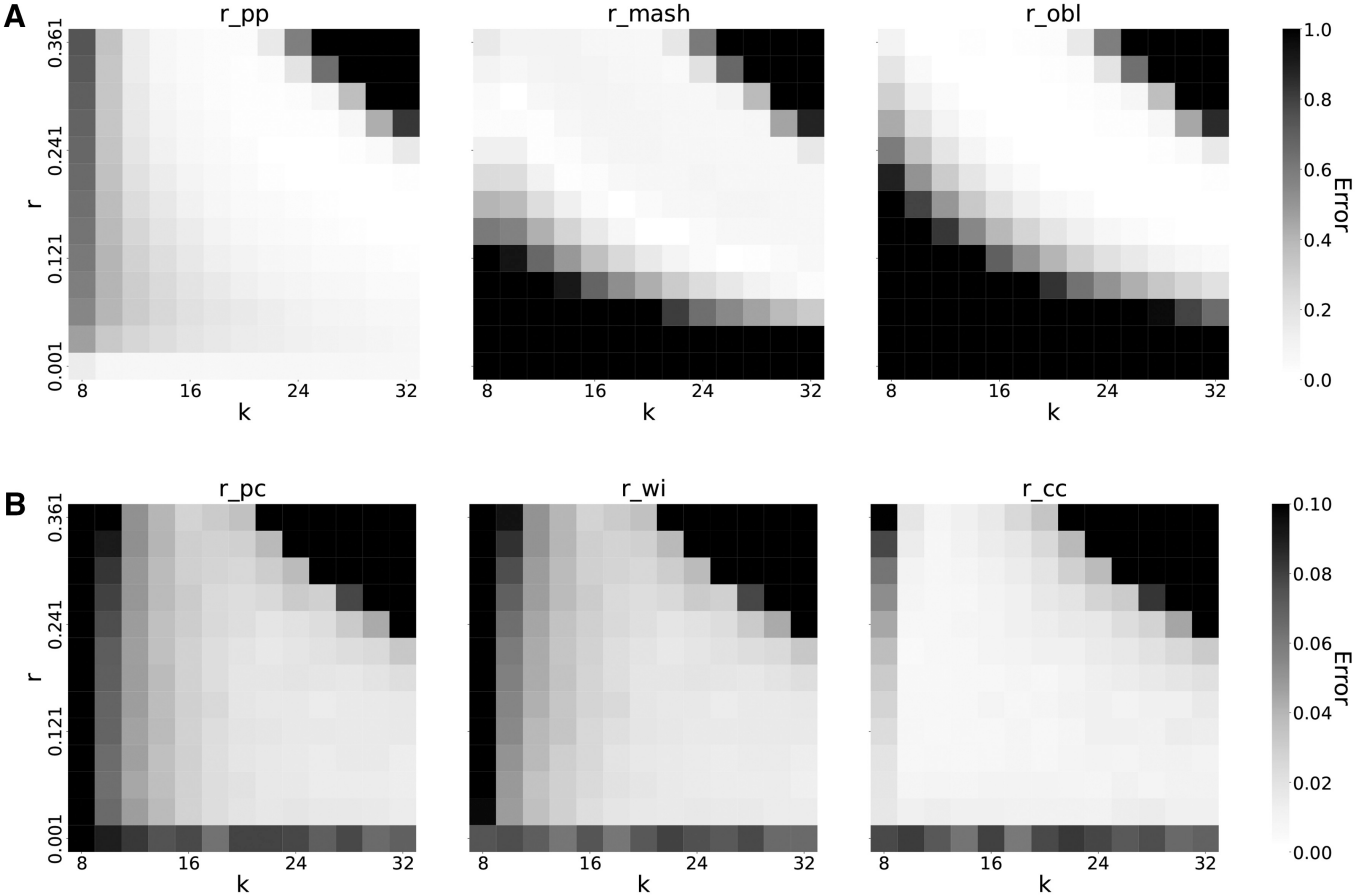
The estimators accuracy on D-hardest as a function of both *k* and *r*. Each cell shows the average relative absolute error of 100 replicates. Note that the scale of the heatmap is different between the top and bottom panels. Moreover, the errors are capped at 1.0 (for A) and 0.10 (for B), e.g. all errors >1.0 are shown as 1.0 in the top panel.

Second, we vary both *k* and *r* and, for each (k,r) pair, compute the average relative absolute error: $\frac{1}{n}\sum_{i=1}^{n}\frac{\left|{\hat{r}}_{i}-r\right|}{r}$ (e.g. Fig. 2 and Table 2). We use n=100 replicates for each (k,r) pair. This error combines the bias and variance into one metric, enabling us to easily visualize accuracy in two dimensions. Note that the two types of benchmarks emphasize different aspects of estimator performance.

**Table 2:** Errors of estimators under different parameter settings.

		r=0.001			r=0.010			r=0.100		
Estimator	Category	k=16	k=24	k=32	k=16	k=24	k=32	k=16	k=24	k=32
${\hat{r}}_{\text{obl}}$	Presence–Presence	202	130	94	19	12	8.7	1.1	0.46	0.18
${\hat{r}}_{\text{mash}}$		14	11	8.9	6.5	4.6	3.5	0.64	0.20	*0.013*
${\hat{r}}_{\text{pp}}$		**0.089**	*0.083*	**0.083**	**0.14**	**0.095**	**0.073**	**0.23**	**0.097**	0.044
${\hat{r}}_{\text{wu}}$		0.88	0.73	0.67	0.30	0.20	0.20	0.37	0.13	0.037
${\hat{r}}_{\text{pc}}$	Presence–Count	0.084	0.084	0.083	0.035	0.029	0.025	0.032	0.018	0.017
${\hat{r}}_{\text{wi}}$	Count–Count	*0.082*	**0.084**	0.081	0.030	0.027	0.024	0.028	0.017	0.017
${\hat{r}}_{\text{cc}}$		0.083	0.085	*0.081*	*0.027*	*0.024*	*0.023*	*0.010*	*0.011*	**0.014**

Each cell shows the average relative absolute error of 100 replicates. Bold values highlight the lowest error in the respective class, while italic values highlight the lowest error overall. Estimator names introduced in the paper are highlighted in bold. The ${\hat{r}}_{\text{wu}}$ estimator falls in between the Presence–Presence and Presence–Count categories.

As observed in our previous work (Wu *et al.* 2025), when *r* and/or *k* become sufficiently large, all *k*-mers mutate with very high probability, causing all tested estimators to return the value 1. We refer to this unstable behavior as *blow-up* and it manifests as high error in the top-right corner of the heatmaps.

### 7.3 Presence–Presence setting

We compare ${\hat{r}}_{\text{pp}}$ with the two other estimators in this category: the estimator defined by Equation (1), which we refer to as ${\hat{r}}_{\text{obl}}$, and the estimator defined by Equation (2), which we refer to as ${\hat{r}}_{\text{mash}}$ (this is the widely used Mash estimator with a binomial correction). Figure 1A shows that for k=30, ${\hat{r}}_{\text{pp}}$ dominates ${\hat{r}}_{\text{mash}}$ across nearly all tested mutation rates; ${\hat{r}}_{\text{pp}}$ dominates ${\hat{r}}_{\text{obl}}$ at lower values of *r* and has similar variance and bias for r>0.161.

Figure 2A presents heatmaps of estimator accuracy across a wide range of *k* and *r*. As expected, all estimators exhibit blow-up behavior for sufficiently large *k* and *r*. However, ${\hat{r}}_{\text{pp}}$ performs substantially better than ${\hat{r}}_{\text{mash}}$ and ${\hat{r}}_{\text{obl}}$ at smaller mutation rates.

### 7.4 Presence–Count and Count–Count setting

For the Presence–Count setting, ${\hat{r}}_{\text{pc}}$ is the only estimator that we are aware of, while for the Count–Count setting, we have our new estimator ${\hat{r}}_{\text{cc}}$ and the ${\hat{r}}_{\text{wi}}$ estimator. Figure 1B shows the performance of these estimators with k=30 and Fig. 2B shows the performance across a wide range of *k* and *r*. Table 2 shows specific values of errors under different settings of *k* and *r*.

First, we note that all estimators in these settings have smaller or similar bias and error than the ${\hat{r}}_{\text{pp}}$ estimator, underscoring the general power of using count information.

Second, in the Count–Count setting, ${\hat{r}}_{\text{cc}}$ outperforms ${\hat{r}}_{\text{wi}}$ for both k=30 and more broadly across most tested (k,r) values. Although ${\hat{r}}_{\text{cc}}$ and ${\hat{r}}_{\text{wi}}$ have access to the same *k*-mer count information, ${\hat{r}}_{\text{cc}}$ achieves nearly unbiased estimation for all tested values of *r* at k=30.

Third, in the Presence–Count setting, ${\hat{r}}_{\text{pc}}$ does not have a direct competitor, so we compare it against ${\hat{r}}_{\text{pp}}$, ${\hat{r}}_{\text{cc}}$, and ${\hat{r}}_{\text{wi}}$. Compared to the ${\hat{r}}_{\text{pp}}$ estimator of the Presence–Presence setting, ${\hat{r}}_{\text{pc}}$ explicitly accounts for the event that multiple *k*-mers can mutate into the same novel *k*-mer, resulting in a smaller bias than ${\hat{r}}_{\text{pp}}$. The estimator ${\hat{r}}_{\text{wi}}$ further addresses certain cases in which a *k*-mer mutates into another *k*-mer already present in the original sequence and, consequently, exhibits a slightly smaller bias than ${\hat{r}}_{\text{pc}}$ for r<0.051. Nevertheless, ${\hat{r}}_{\text{pc}}$ and ${\hat{r}}_{\text{wi}}$ have very similar performance. Compared to ${\hat{r}}_{\text{cc}}$, ${\hat{r}}_{\text{pc}}$ does not perform as well, which is expected since ${\hat{r}}_{\text{cc}}$ is designed to specifically offset some of the bias of ${\hat{r}}_{\text{pc}}$.

### 7.5 Comparison against estimator from Wu *et al.* (2025)

In a previous work, we tackled a similar problem and developed a single repeat-robust estimator (Wu *et al.* 2025). We will refer to it as ${\hat{r}}_{\text{wu}}$ here. It was the first *k*-mer-based estimator evaluated for accuracy in highly repetitive settings, showing robustness in these settings compared to ${\hat{q}}_{\text{obl}}$. It does not neatly fit into the categories here, as it uses the abundance histogram of *s*, i.e. the histogram of *k*-mer occurrence counts. While this is based on the count of the *k*-mers of *s*, it is only a summary and can be approximated using a related species or a related type of sequence. In our framework, therefore, ${\hat{r}}_{\text{wu}}$ lies somewhere between the Presence–Presence and the Count–Presence setting. Figure 1C compares ${\hat{r}}_{\text{wu}}$ against our three estimators for k=30. Table 2 and Table S1 (available as supplementary data at *Bioinformatics* online) show the error across different settings of *k* and *r*.

Despite relying on less information, ${\hat{r}}_{\text{pp}}$ is overall a better estimator than ${\hat{r}}_{\text{wu}}$. The relative bias depends on *r*: ${\hat{r}}_{\text{pp}}$ has slightly smaller bias for 0.001≤r≤0.091 and has slightly larger bias for 0.101≤r≤0.231 (Fig. 1C). However, ${\hat{r}}_{\text{wu}}$ often has more variance than ${\hat{r}}_{\text{pp}}$, as reflected in both Fig. 1C and the bigger relative absolute errors shown in Table 2 and Fig. S1, available as supplementary data at *Bioinformatics* online.

The improvement of ${\hat{r}}_{\text{pc}}$ over ${\hat{r}}_{\text{wu}}$ is more stark, as ${\hat{r}}_{\text{pc}}$ consistently has lower bias than ${\hat{r}}_{\text{wu}}$ (Fig. 1C) and has consistently lower overall error across all values of *k* and *r* (Table 2 and Fig. S1, available as supplementary data at *Bioinformatics* online). The superior performance of ${\hat{r}}_{\text{pc}}$ relative to ${\hat{r}}_{\text{wu}}$ highlights the benefits of accounting for novel *k*-mers in the estimate formula, as ${\hat{r}}_{\text{wu}}$ does not take into account any *k*-mers in *t* that are not in *s*.

### 7.6 Combination with sketching techniques

In Sec. 6, we proved that sketching does change the bias of ${\hat{q}}_{\text{pp}}$ and ${\hat{q}}_{\text{pc}}$. Here, we evaluate empirically the bias of all our three estimators. Figure S2 (available as supplementary data at *Bioinformatics* online) shows the performance of ${\hat{r}}_{\text{pp}}^{\theta }$, ${\hat{r}}_{\text{pc}}^{\theta }$, and ${\hat{r}}_{\text{cc}}^{\theta }$ using θ∈{0.1,0.01}. We observe that sketching does not introduce any systematic bias, in any of the three estimators. As expected with sketching, we observe increased variance for θ=0.1 and even larger variance for θ=0.01. Overall, our results confirm that our estimators can be applied naturally to sketched data, with the obvious caveat that smaller sketches will lead to larger variance of the estimator.

### 7.7 ANI estimation on real genomes

To evaluate the applicability of our estimators on real data, we use $1-{\hat{r}}_{\text{pc}}^{\theta }$ and $1-{\hat{r}}_{\text{cc}}^{\theta }$ to estimate ANI between real genomes. We use the slow but accurate alignment-based OrthoANIu (Yoon *et al.* 2017) to compute the ground truth, consistent with previous work (Shaw and Yu 2023).

We evaluate using a dataset from Hera *et al.* (2023). They first chose ten representative genomes from the Genome Taxonomy Database, including seven bacterial and three archaeal species. For each representative genome, they extract additional genomes from along the evolutionary path to the root, selecting an additional three non-representative genomes at each taxonomic rank on the path up the tree. They construct pairs for comparison by matching each representative genome with the genomes selected along its evolutionary path up the tree. We further filtered out three pairs that had accession numbers that were not currently available. The resulting dataset contains 189 pairs and covers a wide range of ANI from ∼60% to 100%.

We benchmark our estimators against those of of Mash (Ondov *et al.* 2016), sourmash (Irber *et al.* 2024), FastANI (Jain *et al.* 2018), and skani (Shaw and Yu 2023). FastANI and skani rely on mapping techniques with seeds; we run them with default parameters. Sourmash and Mash are both similar to our estimators in that they rely on *k*-mer sketches. We therefore set k=19 for our tools and sourmash and Mash. Sourmash also uses FracMinHash, so we set θ=0.01 for both sourmash and our estimators. Mash uses MinHash sketching instead of FracMinHash, so we set the size of its sketch to be 30,000 to roughly match the sketch sizes obtained with FracMinHash of θ=0.01. Aside from this, we use the default parameters for sourmash and Mash. Table S2 (available as supplementary data at *Bioinformatics* online) gives the full commands and data used.

Figure S3 (available as supplementary data at *Bioinformatics* online) shows the estimator results relative to the ground truth. For some pairs at ANI <85%, some of the estimators either do not report an estimate or report 0; we refer to these as *uncomputable* pairs. In order to make a fair comparison, we measure both the number of uncomputable pairs and the accuracy of the predictions at ANI >85% (Table 3).

**Table 3:** Results on the ANI benchmark.

Tool	n. uncomputable	Pearson	MAE
skani	144	0.9929	0.28
FastANI	128	0.9968	0.19
Mash	11	0.9923	0.40
Sourmash	5	0.9909	0.54
1−${\hat{r}}_{\text{pc}}^{\theta }$	3	0.9904	0.47
1−${\hat{r}}_{\text{cc}}^{\theta }$	3	0.9904	0.47

The number of uncomputable pairs is the number of pairs for which the estimator either returns 0 or does not return anything. Pearson *R* value (Pearson) and mean absolute error (MAE) are shown for a subset of pairs with ANI>85%, to avoid penalizing for uncomputable pairs.

The most accurate estimators at high ANI levels are skani and FastANI; however, they are not able to estimate ANI for more than 100 pairs at lower ANI levels. On the other hand, our estimators are the most comprehensive and are able to compute all except three pairs. Overall, there is a general pattern that more comprehensive estimators are less accurate at high ANI levels, making the choice of the best estimator a trade-off.

## 8 Discussion and conclusion

In this paper, we studied the problem of estimating the mutation rate of a process that transforms an arbitrary string *s* into a string *t* by introducing substitutions at rate *r*. We focused on estimators that are based on *k*-mers, as they can easily be combined with sketching to make them scalable to large datasets. We observed that various estimators for this problem, including our own, can be categorized according to whether they have access to *k*-mer counts or to only presence/absence information. We presented three novel estimators, ${\hat{q}}_{\text{pp}}$, ${\hat{q}}_{\text{pc}}$, and ${\hat{q}}_{\text{cc}}$, summarized in Table 1. On highly repetitive data, ${\hat{q}}_{\text{pp}}$ and ${\hat{q}}_{\text{cc}}$ perform best in their category, with ${\hat{q}}_{\text{cc}}$ outperforming all tested estimators in all categories. The ${\hat{q}}_{\text{pp}}$ estimator is desirable when no count information is available, while the ${\hat{q}}_{\text{pc}}$ estimator offers a middle ground trade-off between accuracy and the amount of information required from the original and mutated sequences.

The main insight behind our ${\hat{q}}_{\text{pp}}$ and ${\hat{q}}_{\text{pc}}$ estimators is that in a repeat setting, it is important to count the *k*-mers that are newly created in *t* and do not appear in *s*. We reflect this in the title, i.e. *novel k*-mers are a *gift* that we must make use of. In many cases, such as ${\hat{q}}_{\text{mash}}$ and ${\hat{q}}_{\text{wu}}$, the formula relies on the observed number of shared *k*-mers. This works fine in the absence of repeats or mutations resulting in spurious matches, as there is a one-to-one correspondence between *k*-mers removed from the intersection and newly created *k*-mers. However, when there are repeats, a mutation in a repetitive *k*-mer τ only destroys one copy of τ and does not remove τ from the count of shared *k*-mers. On the other hand, it does add to the count of new *k*-mers, as $\tau^{\prime }$ is added as a new *k*-mer to *t*. We show that these types of events hurt the performance ${\hat{q}}_{\text{mash}}$ and ${\hat{q}}_{\text{wu}}$, though the effect on ${\hat{q}}_{\text{wu}}$ is compensated by its use of counts. Our ${\hat{q}}_{\text{pp}}$ and ${\hat{q}}_{\text{pc}}$ estimators, on the other hand, are fully based on the count of new *k*-mers, giving them a performance advantage.

In the Count–Count setting, ${\hat{q}}_{\text{wi}}$ already accounts for this insight; in order to out-perform it, we add some accounting for the possibility that a *k*-mer mutates into a *k*-mer that is already in *s*. Consider the possibility that a *k*-mer τ in *s* mutates to a *k*-mer υ while a *k*-mer υ in *s* mutates into τ. The likelihood of this is not directly related to repeats, as it can happen in a repeat-free genome; it is related to two *k*-mers in *s* having a small Hamming distance between them. The ${\hat{q}}_{\text{wi}}$ estimator does not model the chance of this event happening. Our estimator ${\hat{q}}_{\text{cc}}$ models this event happening for the case that the Hamming distance is one; we believe this accounts for its improved performance.

Our mutation model is naturally an idealized version of reality and does not explicitly account for factors such as ploidy or indels. Nevertheless, our estimators can be used as part of downstream tools. For example, the Merqury tool (Rhie *et al.* 2020) computes the quality of a candidate assembly by comparing its *k*-mer content to the *k*-mer content of the sequencing data used to validate it. The score that Merqury reports is closely related to our ${\hat{r}}_{\text{pc}}$ estimator, as it essentially treats the assembly as a mutated string *t* from the original genome *s*. The *k*-mer counts of *t* are given by the assembly; to approximate the presence-absence spectrum of *s*, Merqury uses higher-copy *k*-mers from the read set. Note that what we are describing here differs from the ${\hat{r}}_{\text{wi}}$ estimator, which is also alluded to in Rhie *et al.* (2020).

A natural question arising from our work is how to choose *k* and θ. Guidance on selecting *k* is partially addressed by our heatmaps, which display estimator accuracy across a grid of (k,r) values for both repetitive and typical genomic settings. These plots allow users to identify the stable operating regime for their sequence type. Additionally, in our previous work (Wu *et al.* 2025), we introduced $P_{\text{empty}}$ as a heuristic stability criterion: given sequence length *L*, mutation rate *r*, and *k*, $P_{\text{empty}}$ quantifies the probability that a *k*-mer has no surviving copies after mutation, providing a principled threshold below which the estimator is expected to be unstable. The choice of θ (the sampling rate for sketching) has been widely studied in the sketching literature, though not for our new estimators in particular. Our analysis here (e.g. Fig. S2, available as supplementary data at *Bioinformatics* online) can give a rough guidance for choosing θ, but a more rigorous analysis of sketch estimator stability in repetitive regions remains an interesting problem.

Our insights open the door for the continued improvement of estimators. One particular direction is the Count–Presence setting which we have not discussed in this paper. Note that this setting is not symmetric to the Presence–Count setting; e.g. the counts in *s* are a deterministic variable while the counts in *t* are a random variable. It remains an open problem to determine if estimators in the Count–Presence setting can outperform estimators in the Presence–Count setting. As more repetitive sequences become available, we expect future work to uncover new insights to further drive the improved quality of mutation rate estimators.

## Supplementary Material

btag234_Supplementary_Data

## Data Availability

The benchmark datasets used in this study, including the D-easy, D-med, D-hard, and D-hardest datasets, are available at https://zenodo.org/records/18303511. The ANI benchmark dataset is available at https://github.com/bluegenes/2022-focused-cani-comparisons/blob/main/gtdb-rs207.common-sp10-evolpaths.csv. The software implementation is publicly available at https://github.com/medvedevgroup/Accurate_repeat-aware_kmer_based_estimator.
