# Supplementary material for: The gift of novelty: repeat-robust k-mer-based estimators of mutation rates
Source: Bioinformatics. 2026 Jul 7;42(Suppl 1):btag234. doi: 10.1093/bioinformatics/btag234 (PMC13340239; doi:10.1093/bioinformatics/btag234)
Supplement: btag234_Supplementary_Data [file btag234_supplementary_data.pdf]

A. Supplementary information for "The gift of novelty: repeat-robust k-mer-based estimators of mutation rates" by Haonan Wu and Paul Medvedev

Table S1. Sequence properties of our four datasets. These sequences were originally used for evaluation by Wu et al. (2025). They are extracted from the human T2T-CHM13v2.0 reference and made available to download directly `https://zenodo.org/records/18303511`. The $\overline{d_1}$ column shows the average number of neighbors that have a Hamming distance of 1, i.e. $\overline{d_1} \triangleq \sum_{\tau \in sp(s)} d_1(\tau, s)/|sp(s)|$.

Name	Default k	L	$\|sp(s)\|$	$\overline{d_1}$	Type
D-easy	20	100,000	98,786	0.06	arbitrary region
D-medium	20	14,400	13,727	0.13	RBMY1A gene
D-hard	10	2,264	1,199	0.77	simple repeat
D-hardest	30	100,000	3,987	1.22	centromere

Table S2. Commands used in our ANI benchmark pipeline. The dataset is available for download at `https://github.com/bluegenes/2022-focused-cani-comparisons/blob/main/gtdb-rs207.common-sp10-evolpaths.csv`.

Method (version)	Commands used (arguments in parentheses)
**skani (v0.2.2)**	`skani sketch -t (threads) -o (ref_db) (reference_genomes);`<br>`skani search -d (ref_db) (query_genome) -t (threads) -s (s).`
**Mash (v2.3)**	`mash sketch -o (ref_db/query.msh) -p (threads) -s (sketch_size) -k (k) (reference_genomes);`<br>`mash dist -p (threads) (ref_db/ref.msh) (query_genome).`
**FastANI (v1.33)**	`fastANI -q (query_genome) --rl (ref_list.txt) -o (output.tsv) -t (threads).`
**sourmash (v4.5.0)**	`sourmash sketch dna -p k=(k),scaled=100 --output-dir (ref_db) (reference_genomes);`<br>`sourmash sketch dna -p k=(k),scaled=100 -o (query.sig) (query_genome);`<br>`sourmash search (query.sig) (ref_db/*.sig) -k (k) --max-containment`<br>`--estimate-ani-ci -n 0 -t 0 -o (output.csv).`
**ANIu (v1.2)**	`java -jar (OAU.jar) -n (threads) -f1 (genome1) -f2 (genome2) -u (usearch_binary).`

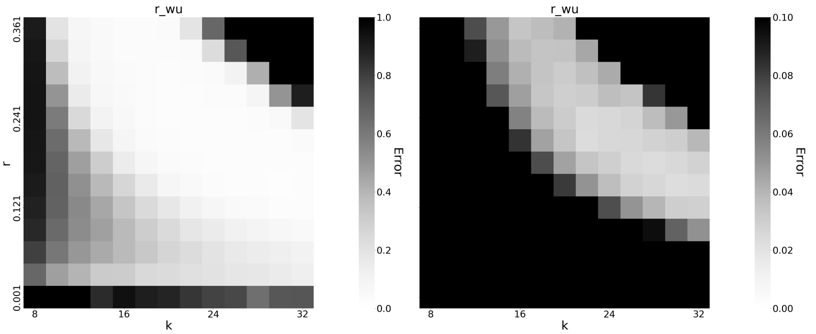

Fig. S1: Heatmaps showing the performance of the $\hat{r}_{\mathrm{wu}}$ estimator on D-hardest. The left heatmap shows the error using a scale up to 1.0, and the right heatmap shows the error using a finer resolution of a scale up to 0.1.

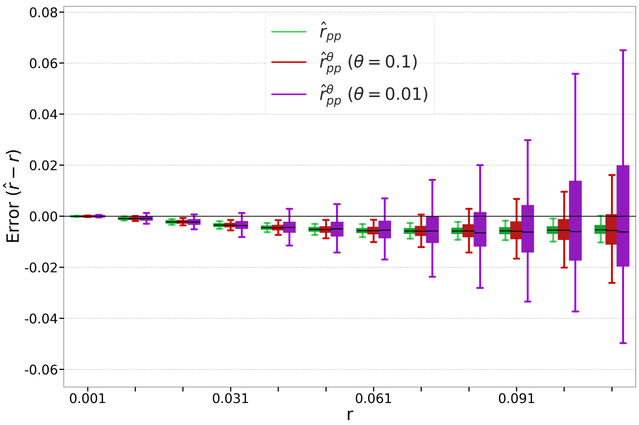

$\hat{r}_{\mathbf{pp}}$

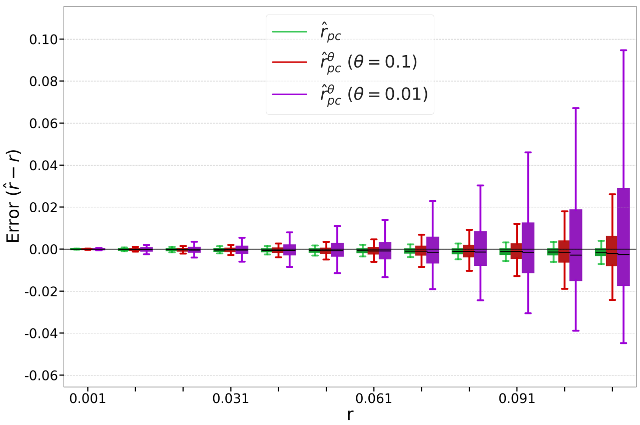

$\hat{r}_{\mathbf{pc}}$

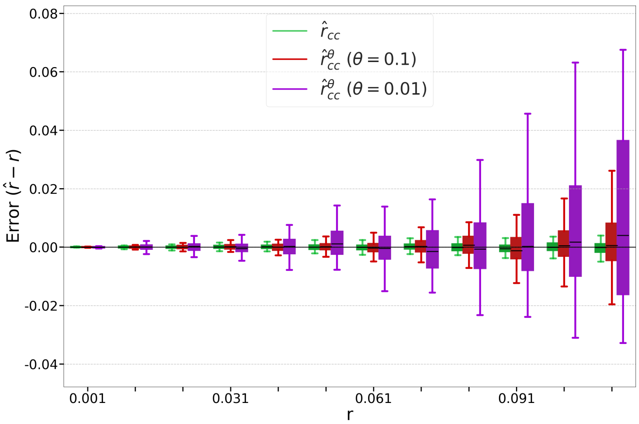

$\hat{r}_{\mathbf{cc}}$

Fig. S2: Effect of sketching on the accuracy of our three estimators, on D-hardest. Top left is $\hat{r}^{\theta}_{\mathrm{pp}}$, top right is $\hat{r}^{\theta}_{\mathrm{pc}}$, and bottom is $\hat{r}^{\theta}_{\mathrm{cc}}$. We vary mutation rates in $r \in [0.001, 0.111]$, using a step size of 0.01. Because sketching introduces additional variance, estimator blow-up occurs at smaller values of r, and we therefore restrict our evaluation to $r \leq 0.111$.

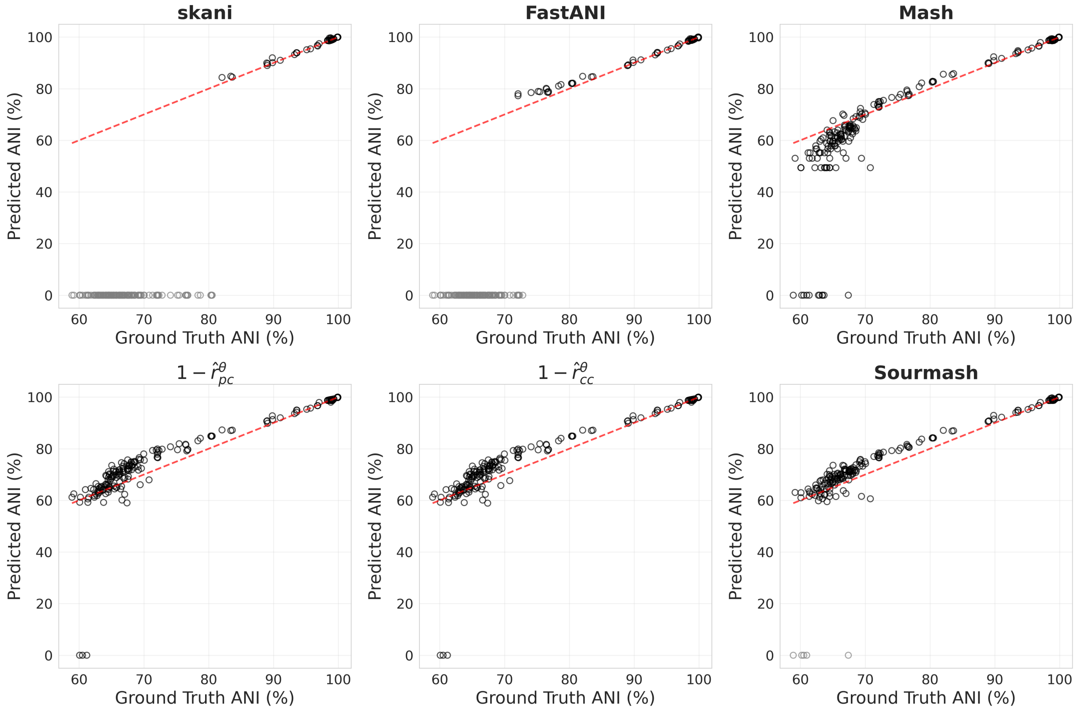

Fig. S3: Performance of various estimators on the ANI benchmark. The dotted line represents a perfect predictor. For genome pairs where the estimator was unable to compute a value, we show the predicted value as 0.

A.1. Comparison of all the estimators on D-easy, D-med, and D-hard

In the main text, we focused on the evaluation of the D-hardest dataset. For completeness, we also include this Supplementary section to show the results on the three easier datasets: D-easy, D-med, D-hard. These datasets were first introduced by Wu et al. (2025) to have progressive levels of repetitiveness. D-easy is an arbitrarily chosen substring of chr6, with less than 1% of k-mers being non-singleton. D-med is the sequence of the chrY RBMY1A1 gene, with approximately 3% of k-mers being non-singleton. D-hard is a subsequence of D-med that is annotated as a simple repeat, with more than 40% of k-mers being non-singletons. These datasets are summarized in Table S1. Fig. S4 shows the results for a single k value and The results are consistent with our findings for D-hardest but the differences become less pronounced on less repetitive sequences. For D-easy and D-med, all the estimators perform relatively well though $\hat{r}_{\mathrm{obl}}$ and $\hat{r}_{\mathrm{mash}}$ show more bias than the rest. For D-hard, the relative performance is similar to that on D-hardest. Fig. S5 shows the heatmaps for a wide range of k and r.

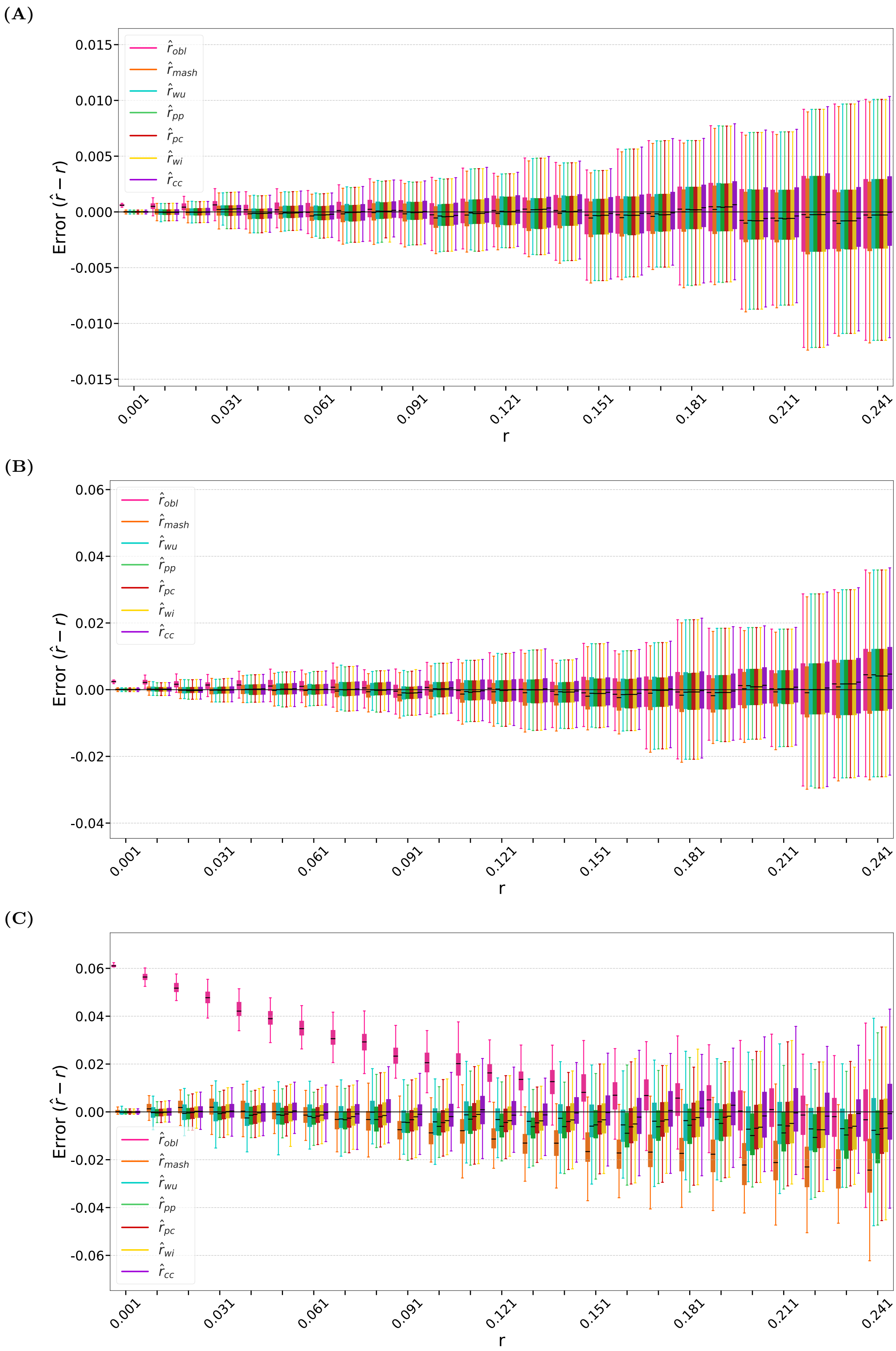

Fig. S4: Comparison of all estimators on the **(A)** D-easy, **(B)** D-med, and **(C)** D-hard datasets. The k values used are as shown in Table S1, i.e. for D-easy, $k = 20$, for D-med, $k = 20$, and for D-hard, $k = 10$.

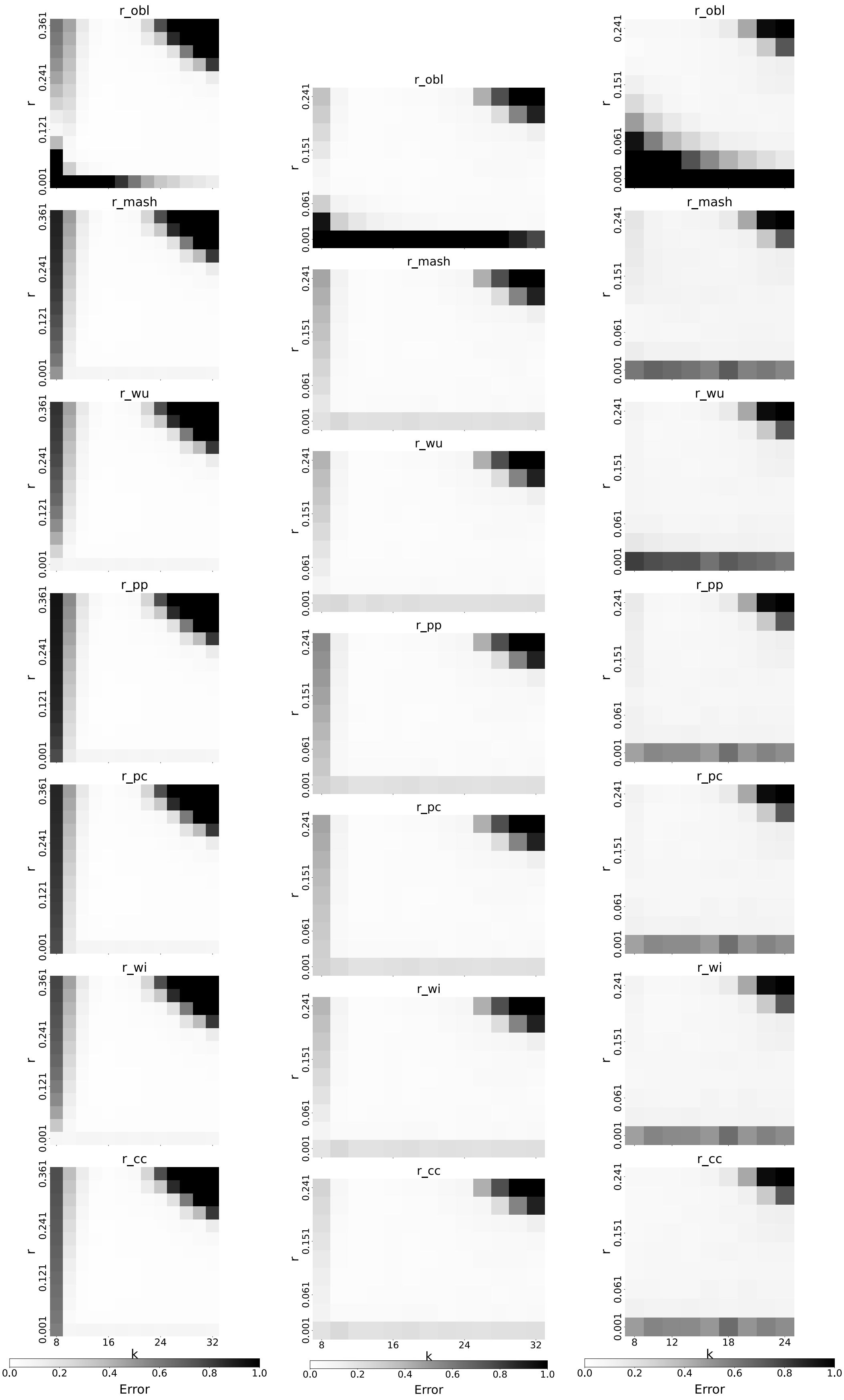

Fig. S5: Heatmap comparison of all estimators on the D-easy (left column), D-med (middle column), and D-hard datasets (right column).
